# AI-Enhanced Deep Learning Framework for Pulmonary Embolism Detection in CT Angiography

**DOI:** 10.3390/bioengineering12101055

**Published:** 2025-09-29

**Authors:** Nan-Han Lu, Chi-Yuan Wang, Kuo-Ying Liu, Yung-Hui Huang, Tai-Been Chen

**Affiliations:** 1 Department of Radiology, E-DA Cancer Hospital, I-Shou University, No. 21, Yida Road, Jiao-Su Village, Yan-Chao District, Kaohsiung 82445, Taiwan; ed102500@edah.org.tw; 2 Department of Medical Imaging and Radiological Science, I-Shou University, No. 8, Yida Road, Jiao-Su Village, Yan-Chao District, Kaohsiung 82445, Taiwan; wang1b011@isu.edu.tw; 3 Department of Radiological Technology, Teikyo University, Tokyo 173-8605, Japan

**Keywords:** pulmonary embolism, CT pulmonary angiography, deep learning, ensemble segmentation, medical imaging, consensus intersection-optimized fusion (CIOF)

## Abstract

Pulmonary embolism (PE) on CT pulmonary angiography (CTPA) demands rapid, accurate assessment, yet small, low-contrast clots in distal arteries remain challenging. We benchmarked ten fully convolutional network (FCN) backbones and introduced Consensus Intersection-Optimized Fusion (CIOF)—a K-of-M, pixel-wise mask fusion with the voting threshold K* selected on training patients to maximize IoU. Using the FUMPE cohort (35 patients; 12,034 slices) with patient-based random splits (18 train, 17 test), we trained five FCN architectures (each with Adam and SGDM) and evaluated segmentation with IoU, Dice, FNR/FPR, and latency. CIOF achieved the best overall performance (mean IoU 0.569; mean Dice 0.691; FNR 0.262), albeit with a higher runtime (~63.7 s per case) because all ten models are executed and fused; the strongest single backbone was Inception-ResNetV2 + SGDM (IoU 0.530; Dice 0.648). Stratified by embolization ratio, CIOF remained superior across <10^−4^, 10^−4^–10^−3^, and >10^−3^ clot burdens, with mean IoU/Dice = 0.238/0.328, 0.566/0.698, and 0.739/0.846, respectively—demonstrating gains for tiny, subsegmental emboli. These results position CIOF as an accuracy-oriented, interpretable ensemble for offline or second-reader use, while faster single backbones remain candidates for time-critical triage.

## 1. Introduction

Pulmonary embolism (PE) is a potentially life-threatening cardiovascular emergency resulting from the obstruction of the pulmonary arteries by embolic material, typically thrombi originating from deep veins in the lower extremities. PE is a major cause of morbidity and mortality worldwide, with an estimated incidence of 60 to 70 cases per 100,000 individuals annually [1]. The clinical presentation of PE is highly variable, ranging from asymptomatic to sudden death, which makes timely diagnosis particularly challenging [2]. Therefore, fast and accurate diagnostic tools are essential to improve patient outcomes and reduce healthcare burden [3].

Computed tomography pulmonary angiography (CTPA or CTA) has become the gold standard for the noninvasive diagnosis of PE [4]. It offers high-resolution cross-sectional imaging of the pulmonary vasculature, enabling the detection of emboli based on contrast-filling defects [5]. Given its widespread clinical use, CTA can be viewed as a modern imaging-based platform capable of capturing spatially and temporally resolved pathophysiological data [6]. However, the manual analysis of CTA images is labor-intensive, time-consuming, and subject to interobserver variability, particularly when emboli are small or located in peripheral branches [7,8,9]. These limitations highlight the need for intelligent image analysis systems that can assist clinicians by providing rapid, consistent, and interpretable segmentation of embolism regions [9].

Recent advances in artificial intelligence (AI), particularly in the domain of deep learning, have revolutionized the field of medical image analysis [10]. Deep learning models, especially convolutional neural networks (CNNs), have demonstrated remarkable performance in various diagnostic tasks, including classification, segmentation, detection, and registration [11,12,13]. Among these, fully convolutional networks (FCNs) have gained significant traction in biomedical image segmentation due to their end-to-end learning capability and pixel-level prediction accuracy [8,14]. FCNs can be trained to automatically detect and delineate complex anatomical or pathological structures in medical images, reducing the reliance on manual annotation and potentially improving diagnostic throughput and accuracy [15,16].

Despite the advantages of FCNs, challenges remain in achieving consistent and generalizable segmentation performance, especially when training data is limited or when model architecture exhibits variability in sensitivity to image features [17,18]. One practical approach to improving robustness is the use of model ensembles, where multiple trained models contribute to the final prediction [3]. Ensemble methods can reduce variance, mitigate overfitting, and leverage the diversity of model outputs to enhance segmentation reliability [19]. Traditional ensemble strategies, such as averaging or majority voting, have been employed in medical imaging studies. However, these methods may not be optimized for semantic overlap, which is crucial in segmentation tasks where alignment with ground truth regions is essential.

To address this limitation, we propose a novel ensemble strategy called Consensus Intersection-Optimized Fusion (CIOF). CIOF is designed to intelligently combine the outputs of multiple FCN models by emphasizing intersection consensus and minimizing false positive inclusion. Rather than relying solely on simple majority voting, CIOF adaptively searches for the voting threshold that yields the maximum intersection with the ground truth while minimizing the union, thus optimizing the Intersection over Union (IoU) metric. This approach emulates signal enhancement strategies in biosensor networks, where multiple sensing signals are fused to improve sensitivity and specificity. CIOF enhances the stability and accuracy of segmentation in complex biomedical images such as CTA scans of pulmonary embolism.

In this study, we applied the CIOF fusion method with ten FCN models trained on the FUMPE dataset, consisting of 2304 CTA slices with expert-annotated PE masks. Five architectures were each trained with Adam and SGDM optimizers. CIOF significantly outperformed individual models in missed detection and segmentation accuracy. This study contributes a comparative evaluation of FCNs, introduces CIOF as an effective fusion strategy, and demonstrates the potential for integrating AI-driven segmentation into imaging-based diagnostic frameworks. The following sections detail the methods, results, discussion, and conclusions.

## 2. The Related Works

### 2.1. Deep Learning-Based Segmentation and Detection of PE

Recent advancements in deep learning have significantly improved the accuracy and efficiency of pulmonary embolism (PE) detection using CT pulmonary angiography (CTPA). Condrea et al. proposed an anatomically aware dual-hop learning framework that integrates anatomical priors into the detection pipeline, enhancing sensitivity to emboli in smaller vessels [2]. Djahnine et al. developed a fully automated 3D deep learning system capable of detecting and quantifying PE severity, demonstrating strong performance across multicenter datasets [4]. Hagen et al. showed that AI algorithms can detect small emboli even in unenhanced CT scans, expanding diagnostic capabilities beyond traditional contrast-enhanced imaging [7].

One of the earliest scalable models, PENet, introduced by Huang et al., utilized volumetric CT data and achieved high diagnostic accuracy across large datasets, setting a benchmark for subsequent deep learning approaches [4]. Kahraman et al. emphasized the importance of segmentation in improving classification performance, demonstrating that integrating segmentation into the pipeline significantly boosts PE detection accuracy [15]. Ma et al. proposed a multitask learning framework that simultaneously performs detection and localization, enabling a more comprehensive analysis of embolic burden [11].

Pan’s work on the RSNA 2020 AI Challenge highlighted the potential of deep learning in real-world competitions, where models were trained on large-scale annotated datasets to detect PE with high precision [9]. Xu et al. introduced Scaled-YOLOv4 for PE detection, leveraging object detection architectures to identify emboli with high speed and accuracy [13]. Yuan et al. focused on pulmonary artery segmentation using PA-Net, incorporating attention mechanisms and contour loss to improve vessel delineation, which is critical for accurate PE localization [14].

These studies collectively demonstrate the versatility of deep learning architectures—from CNNs and multitask models to object detectors and attention-based networking tackling the complex task of PE detection. The integration of anatomical knowledge, 3D volumetric analysis, and segmentation-enhanced classification has led to substantial improvements in diagnostic performance. Moreover, the shift toward multitasking and scalable models reflects a growing emphasis on clinical applicability and robustness. As the field progresses, future models are expected to incorporate multimodal data and real-time inference capabilities, further bridging the gap between research and clinical deployment.

### 2.2. Ensemble, Group Models, and Weak Supervision Approaches

Developments in ensemble learning and weak supervision have significantly enhanced pulmonary embolism (PE) detection performance, particularly in scenarios with limited labeled data or complex imaging features [16]. Abdelhamid et al. introduced a hybrid model combining ResNet50, DenseNet121, and Swin Transformer architectures, achieving high accuracy (97.80%) and AUROC (0.99) by leveraging both convolutional and transformer-based features [16]. This ensemble approach demonstrates the power of integrating diverse model types to capture multi-scale and contextual information in CT pulmonary angiograms.

Belkouchi et al. reported results from the SFR 2022 AI challenge, where multiple teams applied deep learning models to detect and quantify PE severity using standardized datasets [9]. The challenge highlighted the effectiveness of collaborative benchmarking and ensemble strategies in improving generalizability across institutions. Similarly, Biret et al. proposed an integrated deep learning architecture that combines segmentation, classification, and attention mechanisms, resulting in improved detection sensitivity and reduced false positives [17]. Weak supervision has emerged as a promising strategy to reduce annotation burden while maintaining model performance. Hu et al. developed a semi-weakly supervised framework using attention-based CNN–RNN models, showing that only 25% of slice-level labels were sufficient to achieve near-parity with fully supervised models (AUC 0.928 vs. 0.932) [18]. This approach was externally validated on multiple datasets, confirming its robustness and scalability in real-world settings.

Huang et al. demonstrated that automated PE detection using deep learning can be effectively trained on large-scale CT angiogram datasets, even when labels are noisy or incomplete [8]. Their model achieved strong performance metrics and emphasized the importance of data diversity and preprocessing in weakly supervised learning. Vainio et al. further explored transfer learning and open datasets to detect chronic PE from maximum intensity projection images, showing that pretrained models can be fine-tuned effectively for specialized tasks [6].

Together, these studies underscore the value of ensemble modeling and weak supervision in advancing PE detection. By combining multiple architectures, leveraging collaborative datasets, and reducing reliance on exhaustive annotations, researchers are paving the way for scalable, clinically viable AI solutions in radiology.

### 2.3. Systematic Reviews and Broader Surveys

Systematic reviews and broader surveys play a crucial role in synthesizing the rapidly evolving landscape of artificial intelligence (AI) applications for pulmonary embolism (PE) diagnosis. Allena and Khanal provided a comprehensive overview of AI techniques used in PE detection, highlighting the transition from rule-based systems to deep learning models and emphasizing the importance of data quality and interpretability in clinical adoption [16]. Their review also underscored the challenges of integrating AI into routine workflows, including regulatory and ethical considerations. Jabbarpour et al. extended the scope of AI in PE diagnosis by focusing on ventilation/perfusion (V/Q) scintigraphy, a modality often underrepresented in AI literature [19]. Their systematic review traced the evolution of AI tools from early image processing techniques to modern deep learning frameworks, identifying key gaps in dataset availability and validation standards [19]. This work is particularly valuable for expanding AI research beyond CT-based modalities.

Kondamuri et al. offered a broader perspective by reviewing AI applications in chest CT imaging for various lung diseases, including PE [10]. Their analysis categorized models based on architecture, task type, and performance metrics, providing a useful taxonomy for researchers entering the field [10]. While not exclusively focused on PE, the review contextualizes PE detection within the wider domain of thoracic imaging. Abdulaal et al. conducted a focused systematic review on AI tools for chronic PE and chronic thromboembolic pulmonary hypertension (CTEPH), revealing a significant gap in research compared to acute PE [3]. Only a handful of studies addressed chronic PE, and most lacked standardized datasets or direct artery-level assessments, pointing to an urgent need for targeted development in this area [3]. Masoudi et al. contributed to the foundational infrastructure by releasing a publicly available dataset of CT angiography images for PE detection, facilitating reproducibility and benchmarking in future studies [20]. Their work supports the broader survey efforts by enabling standardized evaluation across AI models.

These reviews and surveys provide a panoramic view of AI’s role in PE diagnosis, identifying strengths, limitations, and future directions across modalities, disease stages, and technical approaches.

### 2.4. Clinical Validation, IoMT, and Real-World AI Tools

As artificial intelligence (AI) models for pulmonary embolism (PE) detection mature, clinical validation and real-world integration have become essential for their adoption in healthcare settings. Abed et al. emphasized the importance of workflow-oriented implementation, demonstrating how AI tools can be embedded into radiology pipelines to support PE detection without disrupting clinical routines [21]. Their study highlights the need for seamless integration with PACS systems and radiologist workflows to ensure usability and trust.

Ayobi et al. evaluated an AI-enabled PE detection tool in a clinical setting, reporting high sensitivity and specificity alongside improved diagnostic confidence among radiologists [22]. The study also noted that AI assistance reduced interpretation time, suggesting tangible benefits in emergencies and high-volume environments. Feretzakis et al. extended this validation to COVID-19 patients, where PE detection is particularly challenging due to overlapping pulmonary pathologies [23]. Their deep learning model demonstrated enhanced performance in this subgroup, supporting its utility in pandemic-related care. Grenier et al. developed a deep learning algorithm for automatic PE detection and validated it across multiple institutions, reinforcing its generalizability and robustness [24]. Langius-Wiffen et al. externally validated the RSNA 2020 PE detection challenge-winning model, confirming its performance across diverse datasets and imaging protocols [25]. Such external validation is critical for regulatory approval and clinical deployment.

Lanza et al. introduced a nnU-Net-based model capable of not only detecting PE but also quantifying clot volume and correlating severity with clinical outcomes [26]. This multi-tasking capability enhances the clinical relevance of AI tools, enabling risk stratification and treatment planning. Schmuelling et al. assessed the impact of AI implementation on emergency department workflows, finding no significant change in report turnaround times, which supports the feasibility of AI integration without operational disruption [27]. Vallée et al. demonstrated that AI assistance improves diagnostic accuracy among radiology residents, suggesting a role for AI in education and training [28]. Wiklund and Medson explored AI’s role in triaging incidental PE in cancer patients, showing that deep learning can support early intervention in high-risk populations [29]. Zsarnoczay et al. validated a deep neural network for PE detection across a large multicenter dataset, achieving high accuracy and reinforcing the model’s clinical utility [30].

Collectively, these studies underscore the growing emphasis on clinical validation, workflow compatibility, and real-world performance of AI tools for PE detection. As AI transitions from research to bedside, robust validation and integration strategies will be key to ensuring safe and effective deployment.

## 3. Materials and Methods

**Figure 1:**
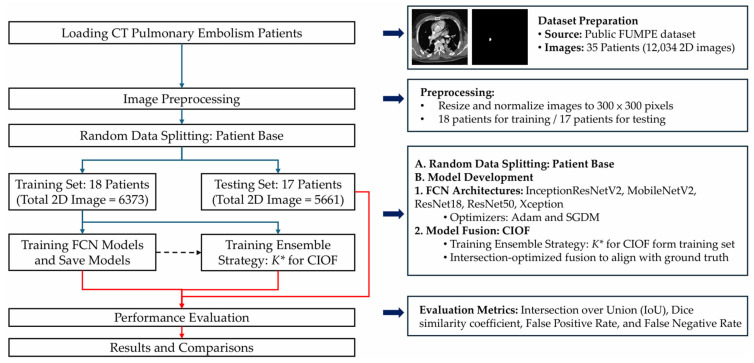
End-to-end CTA-based workflow for pulmonary embolism (PE) detection and segmentation using an ensemble of FCN models. Arrow/key: Solid blue arrows denote the main workflow; the dashed arrow indicates the dependency from the trained FCN models to the K* selection; the red brace marks the evaluation stage on the held-out test set.

To address accurate PE detection and segmentation in CTA, we propose an AI-enhanced deep-learning framework, summarized in Figure 1. The pipeline comprises dataset preparation, patient-level splitting, preprocessing, backbone FCN training, and Consensus Intersection-Optimized Fusion (CIOF). The optimal ensemble threshold K∗ is selected on the training set by maximizing mean IoU (Algorithm 2) and is applied consistently during segmentation (Algorithm 1).

### 3.1. Dataset Description

The dataset used in this study is sourced from a publicly available repository on Kaggle (https://www.kaggle.com/datasets/andrewmvd/pulmonary-embolism-in-ct-images, accessed on 6 March 2025). Known as the FUMPE dataset (Ferdowsi University of Mashhad’s Pulmonary Embolism dataset) [20], it comprises a total of 12,034 computed tomography angiography (CTA) slices collected from 35 patients. Each image is labeled as either Embo (with pulmonary embolism) with a sample size of 2891 or noEmbo (without embolism) with a sample size of 9143. Table 1 shows small emboli (<26 pixels), which typically indicate embolization located in sub-millimeter vessels, are relatively rare, occurring in only 250 slices with a ratio of embolization in images lower than 0.0001.

**Figure 2:**
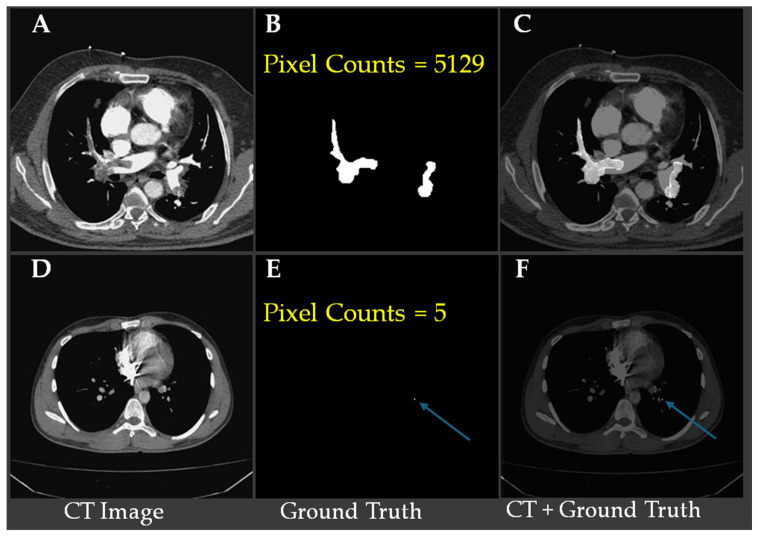
Visual comparison of the largest and smallest (blue arrows) annotated pulmonary embolism regions in CTA images. (**A**) CT image with a large PE region; (**B**) Ground-truth mask for (**A**); pixel count 5129; (**C**) Overlay of (**A**) and (**B**) (CT + ground truth); (**D**) CT image with a very small PE region; (**E**) Ground-truth mask for (**D**); pixel count 5 (blue arrow); (**F**) Overlay of (**D**) and (**E**); blue arrows indicate the tiny PE.

Figure 2 shows representative CTA slices illustrating the largest and smallest annotated pulmonary embolism regions. Figure 2A–C show a case with the largest embolic region (5129 pixels), where (Figure 2B) displays the ground truth segmentation and (Figure 2C) overlays the embolism on the CT slice. Figure 2D–F demonstrate the smallest annotated embolism in the dataset, consisting of only five pixels, with the location indicated by blue arrows in (Figure 2E,F). These examples underscore the substantial variability in embolism size and highlight the challenge of detecting minute emboli, which requires high model sensitivity and precise localization to avoid false negatives.

### 3.2. Image Preprocessing

Image intensities were normalized using Hounsfield Unit (HU) windowing with brain or lung windows depending on model input strategies. For models requiring fixed input dimensions, 2D axial slices were extracted with a size of 300 × 300 pixels. Data augmentation techniques, including random rotations, flipping, intensity jittering, and elastic deformation, were applied to enhance generalizability.

### 3.3. Model Architectures

The proposed workflow (Figure 1) integrates fully convolutional networks (FCNs) with a decision-level fusion strategy for robust pulmonary embolism (PE) segmentation. All computed tomography angiography (CTA) images are first resized to a standardized resolution of 300 × 300 × 3. Each image is then converted into an RGB composite using three input channels—bone window, brain window, and their average—to enhance contrast and improve vascular structure visualization for better clot localization.

A total of five pre-trained semantic segmentation backbones is employed in the FCN framework: InceptionResNetV2, Xception, MobileNetV2, ResNet50, and ResNet18. Each backbone is trained separately with two optimization strategies—ADAM and Stochastic Gradient Descent with Momentum (SGDM)—resulting in a total of 10 model variants. Each model independently produces a binary segmentation mask to localize embolism regions (EMBO class) from the input CTA slices. The predicted probability maps are thresholded to generate binarized segmentation masks.

To quantify model contribution to fusion, we define the Consensus Intersection-Optimized Fusion (CIOF) map as shown in Algorithm 1. *TP* represents the number of pixels that exceed the consensus optimal threshold K*.
**Algorithm 1: Consensus Intersection-Optimized Fusion (CIOF)**Input: Dataset D, FCN models results {M_1_, M_2_, …, M_10_}, consensus optimal threshold K* Output: Final fused mask CIOF *1: Load dataset D and models {M_1_*, …, *M_10_}* *2: For each patient p in D do* *3:          For i = 1 to 10 do* *4:                  Mask_i_ = M_i_(p) # segmentation result of ith FCN* *5:          End For* *6:          *$SumMask=\sum_{i=1}^{10}Mask_{i}$ *7:          *$CIOF=1\left\{SumMask>K^{*}\right\}$ 8: End For *9: Return CIOF*

*Mask_i_* is the segmentation result (binary mask) produced by the *i*th FCN model for image I. *SumMask* is the joint boundary map obtained by summing the predictions of all 10 FCN models; *TP* is the majority voting boundary. K* is optimized on the training set using fusion of masks generated by ten FCN models, with IoU (Intersection over Union) as the maximization criterion, as shown in Algorithm 2. *|D|* is the number of patients in dataset D.
Algorithm 2: Training Selection of the Optimal Voting Threshold K* for CIOF*Input: Dataset D using training patients only, FCN models {FCN_1_, FCN _2_, …, FCN _10_}* Output: Optimal threshold K* for testing set *1: Load dataset D and models {FCN_1_*, …, *FCN _10_}* *2: For k = 1 to 10* *3:          For each patient p in D do # Patient index* *4:                    For i = 1 to 10 do # FCN model Index* *5:                            Mask_i_ = M_i_(p) # segmentation result M_i_(p) by FCNi* *6:                  End For* *7:                  *$SumMask=\sum_{i=1}^{10}Mask_{i}$ *8:                  *$Mask=1\left\{SumMask\geq k\right\}$ *9:                  Computed* $IoU_{p}$ *using Mask and ground truth label* *10:            End For* *11:            *$IoU_{k}=\sum_{p=1}^{\left|D\right|}IoU_{p}/|D|$ *12: End For* *13: Return* $K^{*}=\underset{k}{\mathrm{argmax}}{\{IoU}_{k},k=1,\ldots ,10\}$

To enhance robustness and reduce false positives, we use a consensus-based ensemble (CIOF). Each FCN outputs a binary mask; masks are aggregated pixel-wise, and a pixel is labeled embolus if at least *K* of M models votes positive. In this study, M = 10, and the threshold K* is selected on training patients only by maximizing IoU (empirically, K* = 2). All data splits and model-selection steps are strictly patient-based—no slices from the same patient appear in more than one partition. This fusion improves the detection of small or low-contrast emboli while suppressing idiosyncratic false alarms. CIOF also yields per-model agreement scores, indicating each model’s alignment with the fused result; higher values reflect stronger contributions to the final decision, which is particularly informative for difficult-to-localize clots (see Figure 2E). The modular design balances segmentation accuracy, interpretability, and clinical applicability while remaining flexible for alternative fusion rules or retraining strategies.

### 3.4. Training Details

All models were trained using the Adam and SGDM optimizers with an initial learning rate of 1 × 10^−4^ and a batch size of 30. Data partitioning followed a patient-level protocol. The FUMPE cohort (35 patients; 12,034 axial CTA slices) was randomly divided into a training set of 18 patients (6373 slices) and a test set of 17 patients (5661 slices). All model fitting and ensemble threshold selection (K* for CIOF) were performed only on training patients; the test patients were held out for final evaluation. This patient-based design eliminates slice-level leakage and reflects real-world deployment. The loss functions included Dice Loss for segmentation, Binary Cross-Entropy for classification, and a composite loss (Dice + Focal) for class-imbalanced scenarios. Training was conducted for 500 epochs with early stopping based on validation Dice coefficient.

The full set of training hyperparameters is reported below. We evaluated SGDM (momentum = 0.9) and Adam (β_1_ = 0.9, β_2_ = 0.999). A momentum of 0.9 is a widely adopted default in CNN-based segmentation and yields stable convergence on this small medical dataset. Learning rate and schedule: we used an initial LR of 1 × 10^−4^; after testing a step schedule (drop factor 0.1 every 10 epochs), a constant LR produced equal or better validation Dice and was therefore retained. L2 regularization was grid-searched over {1 × 10^−5^, 1 × 10^−4^, 5 × 10^−4^}; 1 × 10^−4^ best balanced overfitting control and optimization speed on the FUMPE cohort. The gradient threshold method is set as L2-Norm with no clipping because losses and gradients were stable; enabling clipping did not materially change the results. The execution environment applied GPU execution to the specified hardware (RTX 3090 Ti) for both training and inference. When a held-out validation set was available, training was used early, stopping with a maximum of 500 epochs, a validation frequency of 50 iterations, and a validation patience of five epochs; the checkpoint corresponding to the lowest validation loss was retained. This standard early-stopping protocol helps limit overfitting on small datasets.

All experiments were conducted using MATLAB R2023a with the Deep Learning Toolbox with CUDA 12.1. The computational setup consisted of an AMD Ryzen Threadripper PRO 3995WX processor (64 cores, base clock 2.70 GHz, boost up to 3.57 GHz), 256 GB DDR4 RAM, an NVIDIA GeForce RTX 3090 Ti GPU with 24 GB VRAM, and the Windows 11 Pro 64-bit operating system. This configuration was used for training all CNN models.

### 3.5. Evaluation Metrics

Model performance was quantitatively evaluated using several standard metrics, including the Intersection over Union (IoU), Dice coefficient, false negative rate (FNR), and false positive rate (FPR), as defined in Equations (1)–(4).

All evaluations were performed on the held-out test patients. For a binary prediction P and ground truth G on a slice, define TP = ∣P∩G∣, FP = ∣P∖G∣, FN = ∣G∖P∣, and TN as the remaining pixels.

(1) $$IoU=\frac{\left|P\cap G\right|}{\left|P\cup G\right|}=\frac{TP}{TP+FP+FN}$$

(2) $$Dice=\frac{2|P\cap G|}{\left|P\right|+|G|}=\frac{2TP}{2TP+FP+FN}$$

(3) $$FNR=\frac{FN}{TP+FN}$$

(4) $$FPR=\frac{FP}{FP+TN}$$

Majority voting maps are binarized at a fixed threshold (*K* = 5 unless stated). CIOF fusion uses a fixed consensus threshold K*, chosen only on training patients and kept unchanged for testing (K* = 2). Where noted, metrics are additionally stratified by embolization burden (e.g., per-slice embolization ratio <10^−4^, 10^−4^–10^−3^, >10^−3^) and by embolus size in pixels (<26, 26–262, >262). Computational cost is reported as the mean inference time per case (seconds per study).

## 4. Results

This section presents a comprehensive evaluation of the segmentation performance of various deep learning models and the proposed CIOF method for pulmonary embolism (PE) detection in CTA images. The analysis is stratified by embolization ratios to assess model sensitivity under varying clot burdens. Key performance metrics, including the Dice coefficient and Intersection over Union (IoU), are reported to quantify segmentation accuracy. In addition, small sizes of emboli are analyzed to understand clinical applicability, especially in challenging scenarios involving small emboli. The results highlight both the effectiveness and limitations of each method, providing insights into their diagnostic value.

### 4.1. The Optimal K* for CIOF

**Figure 3:**
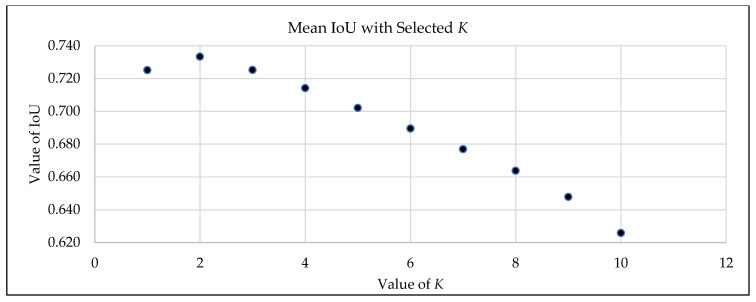
Selection of the optimal voting threshold K* for CIOF on the training patients.

To determine the optimal consensus threshold K* for the CIOF ensemble, we trained M = 10 FCN models on the training patients and evaluated thresholds *K* ∈ {1, …, 10}. For each *K*, fused masks were created by labeling a pixel as embolus when at least *K* models voted positive; IoU with ground truth was computed per slice, averaged between the patients, and then averaged across the training cohort. As shown in Figure 3, the mean IoU is highest at *K* = 2 (≈0.74) and declines for a larger *K*, reflecting increasing false negatives under stricter consensus; *K* = 1 is more permissive and yields more false positives. We therefore fixed K* = 2 and used this unchanged value for all evaluations on the held-out test patients, preventing information leakage and aligning the ensemble with the consensus behavior of the individual models. The Majority Vote is applied as *K* > 5 in this study.

### 4.2. The Performance of Segmentation of Models

Table 2 summarizes accuracy and runtime (mean inference time per case, in seconds) across ten FCN backbones and two ensemble schemes. The CIOF ensemble delivers the best overall accuracy (IoU = 0.569, Dice = 0.691) and the lowest FNR (0.262), indicating superior sensitivity to emboli—including small or low-contrast regions. This comes with a higher FPR (0.169) and the largest latency (≈63.7 s) because all 10 models are executed and fused.

Among single backbones, Inception-ResNetV2 + SGDM performs best (IoU = 0.530, Dice = 0.648) with a moderate runtime (~15.1 s). The ResNet50 variants offer a strong accuracy–latency trade-off (Dice ≈ 0.563–0.564, ~5.3 s) and thus are attractive for near real-time use. ResNet18 is the fastest (~2.8–2.9 s), and MobileNetV2 is also quick (~4.0 s), but both trade speed for lower accuracy (Dice ≤ 0.539). The Majority Vote baseline yields intermediate runtime (≈63.7 s) and markedly worse accuracy (IoU = 0.373, Dice = 0.482) despite an extremely low FPR (0.014), reflecting an overly conservative decision rule that misses many emboli (FNR = 0.613). The weakest results are from Xception + SGDM (IoU = 0.195, Dice = 0.271).

CIOF provides the top segmentation accuracy and sensitivity, suitable when detection completeness is paramount; ResNet50 + SGDM (or Inception-ResNetV2 + SGDM when slightly higher accuracy is desired) offers the best accuracy–latency balance for time-constrained workflows.

### 4.3. The Impact of Clinical Applications by Using CIOF

Table 3 shows the detection capability of different models across varying embolization burdens, which were stratified into three categories: <0.0001, 0.0001–0.001, and >0.001 (Table 3). To assess clinical robustness, slices were stratified by embolization ratio—the fraction of embolus pixels per slice—into three ranges: <10^−4^, 10^−4^–10^−3^, and >10^−3^. Performance rose monotonically with an increasing clot burden for all methods, yet the CIOF ensemble remained the top performer in every stratum, achieving a mean IoU/Dice of 0.238/0.328 for tiny emboli (<10^−4^), 0.566/0.698 for a medium burden (10^−4^–10^−3^), and 0.739/0.846 for a large burden (>10^−3^). Relative to the strongest single-model baseline (Inception-ResNetV2 + SGDM), CIOF improved IoU by 0.025, 0.044, and 0.025 across the three strata, respectively; the margins over Majority Vote were still larger (+0.209, +0.219, +0.107 IoU).

Majority Vote was particularly conservative for tiny emboli (0.029/0.043 IoU/Dice), improving only when clot burden increased (0.632/0.764 for >10^−3^), while representative single backbones such as ResNet50 + SGDM followed the same trend (0.169/0.234, 0.426/0.547, 0.666/0.784). Collectively, these results show that although segmentation accuracy improves with embolic extent for all models, CIOF provides the greatest advantage where detection is most challenging (the <10^−4^ stratum) while maintaining state-of-the-art accuracy at moderate and large burdens, supporting its suitability across diverse PE presentations.

## 5. Discussion

### 5.1. Effectiveness of CIOF in Segmenting Pulmonary Emboli in CT Angiography

Small pulmonary emboli, particularly those occupying fewer than 26 pixels in CT angiography (CTA) images, are frequently located in the distal branches of the pulmonary arteries, including subsegmental or microvascular regions. Although these emboli contribute to a smaller overall clot burden, their detection remains clinically important due to several factors:

- Clinical Risk in Vulnerable Patients: In patients with comorbidities (e.g., cancer, thrombophilia), even small emboli may lead to adverse outcomes due to impaired pulmonary perfusion.
- Challenge for AI Models: Small emboli pose a technical challenge due to their low contrast, small size, and location in vessels close to image resolution limits. Models must exhibit high sensitivity and precise localization.

A single pixel in CTA typically represents 0.5–0.7 mm depending on resolution. Therefore, an embolus <26 pixels corresponds to a clot size spanning ~13 mm^2^, likely involving subsegmental or smaller branches (Table 4). CIOF might have potential to detect small spots of emboli in CT image.

This result underscores the utility of ensemble-based fusion strategies in handling the “long tail” of difficult cases—such as microvascular emboli—where both anatomical complexity and imaging constraints coexist. By increasing sensitivity without sacrificing specificity, CIOF provides a clinically promising path forward for integrating deep learning into AI-assisted radiology workflows.

**Figure 4:**
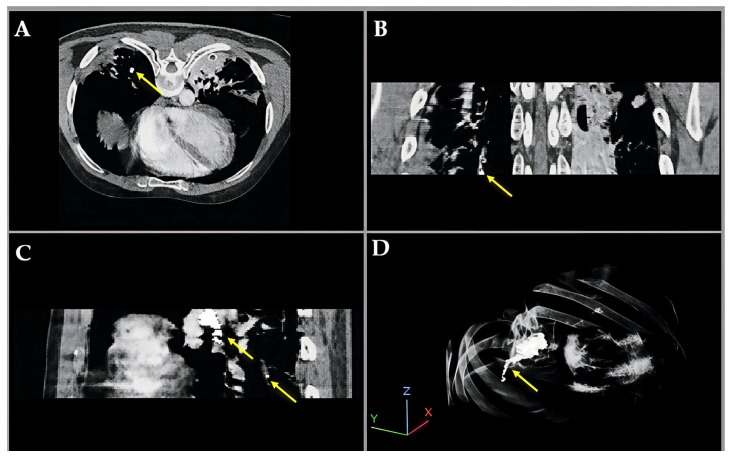
Multiview visualization of pulmonary embolism (PE) in CT angiography, illustrating enhanced intensity and fused labeling between the CT image and segmentation results. (**A**) Axial (XY) view; (**B**) coronal (XZ) view; (**C**) sagittal (YZ) view; and (**D**) 3D volumetric reconstruction.

Figure 4 illustrates a pulmonary embolism (PE) detected in a patient’s CT angiography, shown across axial, coronal, sagittal, and 3D views. Yellow arrows highlight the embolus location within the pulmonary artery, demonstrating the value of multi-planar visualization in accurately assessing PE size and position.

### 5.2. Robust Segmentation of Subsegmental Emboli with CIOF Fusion

Subsegmental emboli (SSE) reside in distal pulmonary arterial branches where vessel caliber approaches the in-plane CTA resolution; consequently, target regions are small, low-contrast, and susceptible to partial-volume effects and motion artifacts. These factors cause single FCN backbones to exhibit model-specific blind spots—typically manifesting as fragmented masks or outright misses. The proposed CIOF fusion counters these failure modes by aggregating pixel-wise votes from M = 10 independently trained FCNs and labeling a pixel as embolus when at least K* = 2 models agree (selected on training patients to maximize IoU). This light-consensus threshold strengthens weak but spatially coherent signals while suppressing idiosyncratic false alarms, yielding a mask that is both more sensitive to tiny clots and more topologically consistent along the vessel path.

Empirically, CIOF delivers the highest segmentation accuracy for slices dominated by SSE. In the small-burden stratum (<10^−4^ embolization ratio), CIOF achieves IoU/Dice = 0.238/0.328, outperforming the strongest single model, Inception-ResNetV2 + SGDM (0.213/0.293), and far exceeding the conservative Majority Vote baseline (0.029/0.043) that frequently misses small clots (Table 3). Performance gains persist at a medium burden (10^−4^–10^−3^; 0.566/0.698 vs. 0.522/0.648) and large burden (>10^−3^; 0.739/0.846 vs. 0.714/0.823), indicating that CIOF improves sensitivity without sacrificing precision as the clot extent increases. Consistent with Table 2, CIOF also yields the lowest FNR (0.262) among all methods, highlighting fewer missed detections than those of other methods in this study.

From a deployment perspective, CIOF’s robustness comes at the cost of higher latency (ensemble inference and fusion). This trade-off is acceptable for patient-based offline analysis, second-reader support, or quality-assurance workflows, while faster single backbones (e.g., ResNet50 + SGDM) can serve as time-critical first passes. CIOF provides sensitivity for subsegmental disease with interpretable consensus behavior (via per-model CIOF agreement scores), strengthening its suitability for detecting subtle or early presentations of pulmonary embolism in routine CTA.

### 5.3. Comparative Evaluation of CIOF Against State-of-the-Art PE Segmentation Approaches

Table 5 summarizes the performance of recent deep learning approaches for pulmonary embolism (PE) detection and segmentation using CT pulmonary angiography (CTPA). Xu et al. (2023) [8] applied a detection-focused model, Scaled-YOLOv4, and reported variable results across datasets, with Tianjin achieving a Dice of 0.930 but a FUMPE only 0.7274, indicating dataset dependency. Pu et al. (2023) [31] used an R2-Unet without manual outlining on the RSNA PE dataset and obtained a Dice of 0.676, showing automation potential but lower accuracy compared to ensemble-based methods. Chu et al. (2025) [32] achieved high performance (Dice = 0.918) by segmenting pulmonary arteries and veins from multi-center data, suggesting strong generalizability.

Advanced architecture has further pushed segmentation accuracy. Liu et al. (2022) [33] introduced CAM–Wnet, achieving an IoU of 0.935 on the China–Japan Friendship Hospital dataset, while Nitha et al. (2025) [34] reported Dice = 0.94 with a two-stage pipeline. Region-based methods also demonstrated superior outcomes: Doğan et al. (2024) [35] achieved the highest Dice (0.95) with an enhanced Mask R-CNN approach, highlighting the advantages of instance-level segmentation. By comparison, Fan et al. (2025) [36] reached Dice = 0.8257 using a threshold adjustment segmentation network applied across six hospitals, reflecting clinical scalability but slightly lower accuracy than R-CNN or CAM–Wnet.

The proposed CIOF ensemble, integrating ten fully convolutional networks (FCNs), achieved a Dice score of 0.846 on the FUMPE dataset, with emboli sizes > 0.001 (>262 pixels). This performance surpasses many single-network methods and approaches the accuracy of advanced region-based and multi-stage models. Importantly, CIOF demonstrates consistent detection across embolization ratios, particularly excelling in small emboli cases where other models show limitations. This suggests that while Mask R-CNN and CAM–Wnet variants may offer higher peak Dice values, CIOF provides a balanced and robust solution optimized for both segmentation accuracy and clinical applicability.

## 6. Conclusions

This study presents CIOF (Consensus Intersection-Optimized Fusion), an ensemble that aggregates predictions from ten fully convolutional networks to improve pulmonary embolism (PE) segmentation on CT angiography using patient-based splits. CIOF achieved a mean Dice of 0.691 on the held-out test cohort. Stratified by clot size, Dice was 0.328 for small (<26 pixels), 0.698 for moderate (26–262 pixels), and 0.846 for large emboli (>262 pixels). The mean ensemble inference time was 63.7 s per case (all ten models). These results position CIOF as an accuracy-oriented, interpretable ensemble for offline or second-reader use, while faster single backbones remain candidates for time-critical triage.

## 7. Limitations and Future Works

While the proposed CIOF framework demonstrates high segmentation accuracy and robust detection performance across various embolization levels, several limitations should be acknowledged.

First, this study was conducted on the FUMPE single-center cohort comprising 35 patients, which may limit the generalizability of the results across institutions with different scanner types, imaging protocols, or population demographics. Second, although the ensemble approach effectively boosts performance, it also increases computational complexity and may hinder real-time deployment in clinical environments without GPU acceleration. To mitigate this, all models in this present study were trained offline, and inference was parallelized across GPUs. For future clinical deployment, lightweight ensemble strategies or model distillation approaches will be investigated to reduce inference time while retaining accuracy. In addition, lesion-level sensitivity/specificity (e.g., FROC) and patient-level ROC analysis were not performed. Also, several further limitations warrant consideration.

For future work, we plan to (1) validate the CIOF model on multi-center and multi-vendor datasets to ensure broader applicability; (2) investigate lightweight ensemble strategies or model distillation to reduce inference time; (3) incorporate clinical metadata and anatomic priors to improve interpretability and diagnostic relevance; (4) re-organize datasets using patient-specific splits to eliminate cross-patient leakage and provide more rigorous evaluation; (5) extend the framework toward weakly supervised and semi-supervised learning paradigms to better leverage limited labeled data; particularly in cases involving small or low-contrast emboli; (6) investigate advanced 3D deep learning architectures [37,38] to enhance volumetric feature representation; and (7) integrate CAMs or other explainable AI techniques to improve transparency and clinical interpretability.

## Figures and Tables

**Table 1:** Distribution of pulmonary embolism sizes by pixel count and ratio of embolization in image.

Pixel Count of Embolization	Ratio of Embolization in Image	N
<26	<0.0001	250
26~262	0.0001~0.001	1977
>262	>0.001	664

**Table 2:** Quantitative comparison of segmentation performance across different FCN architectures and the proposed CIOF method.

Methods	Mean IoU	Mean Dice	Mean FNR	Mean FPR	Mean Inferred Times (s)
CIOF	0.569	0.691	0.262	0.169	63.7
Majority Vote	0.373	0.482	0.613	0.014	63.7
InceptionResNetV2 + Adam	0.390	0.499	0.479	0.132	14.9
InceptionResNetV2 + SGDM	0.530	0.648	0.388	0.082	15.1
MobileNetV2 + Adam	0.423	0.539	0.421	0.157	4.0
MobileNetV2 + SGDM	0.327	0.435	0.589	0.083	4.0
ResNet18 + Adam	0.394	0.504	0.542	0.064	2.8
ResNet18 + SGDM	0.341	0.453	0.632	0.026	2.9
ResNet50 + Adam	0.445	0.564	0.470	0.084	5.3
ResNet50 + SGDM	0.449	0.563	0.505	0.046	5.3
Xception + Adam	0.302	0.398	0.653	0.046	4.8
Xception + SGDM	0.195	0.271	0.710	0.095	4.7

**Table 3:** Stratified segmentation performance across embolization ratios.

Methods	Ratio of Embolization in Image					
	<0.0001		0.0001~0.001		>0.001	
	Mean IoU	Mean Dice	Mean IoU	Mean Dice	Mean IoU	Mean Dice
CIOF	0.238	0.328	0.566	0.698	0.739	0.846
Majority Vote	0.029	0.043	0.347	0.463	0.632	0.764
InceptionResNetV2 + Adam	0.059	0.083	0.371	0.484	0.617	0.753
InceptionResNetV2 + SGDM	0.213	0.293	0.522	0.648	0.714	0.823
MobileNetV2 + Adam	0.090	0.133	0.404	0.528	0.649	0.780
MobileNetV2 + SGDM	0.066	0.098	0.308	0.417	0.524	0.664
ResNet18 + Adam	0.086	0.119	0.365	0.480	0.648	0.779
ResNet18 + SGDM	0.091	0.127	0.325	0.440	0.523	0.660
ResNet50 + Adam	0.128	0.178	0.436	0.560	0.633	0.766
ResNet50 + SGDM	0.169	0.234	0.426	0.547	0.666	0.784
Xception + Adam	0.024	0.035	0.269	0.363	0.555	0.699
Xception + SGDM	0.031	0.045	0.174	0.245	0.348	0.474

**Table 4:** Pulmonary vessel diameter map (for illustration/explanation).

Vessel Type	Approx. Diameter	Notes
Main pulmonary artery	20–25 mm	Arises from right ventricle
Lobar arteries	8–10 mm	First bifurcation
Segmental arteries	4–6 mm	Supplies lung segments
Subsegmental arteries	2–3 mm	Supply secondary divisions
Intrapulmonary arterioles	0.5–1.5 mm	May be visible in high-res CTA
Capillary-level Micro vessels	<0.1 mm	Beyond CTA resolution—emboli here are inferred indirectly

**Table 5:** Comparative analysis of recent deep learning approaches for pulmonary embolism (PE) detection and segmentation using CT pulmonary angiography (CTPA). Performance is reported using Dice or IoU when available.

Author (Year)	Method	Dataset	Dice/IoU	
Xu et al. (2023) [13]	Scaled-YOLOv4	Tianjin, Linyi, and FUMPE datasets	Tianjin Dice = 0.930 Linyi Dice = 0.759 FUMPE Dice = 0.727	
Pu et al. (2023) [31]	R2-Unet	RSNA pulmonary embolism CT dataset	Dice = 0.676	
Chu et al. (2025) [32]	High-abundant pulmonary artery-vein segmentation	Multi-center dataset	Dice = 0.918	
Liu et al. (2022) [33]	CAM–Wnet architecture	China–Japan Friendship Hospital dataset	IoU = 0.935	
Nitha et al. (2025) [34]	A two-stage deep learning pipeline	Aster Medcity multi-speciality hospital, Kochi	Dice = 0.940	
Doğan et al. (2024) [35]	An enhanced mask R-CNN approach	Radiology department of Kahramanmaraş Sutcu Imam University	Dice = 0.950	
Fan et al. (2025) [36]	Threshold adjustment segmentation network	Six different hospitals	Dice = 0.826	
The Presented Method (2025)	Ensemble of 10 FCNs	FUMPE	Mean Dice = 0.691	
			Ratio of Embolization	Mean Dice
			<0.0001	0.328
			0.0001~0.001	0.698
			>0.001	0.846

## Data Availability

The CT PE datasets used in this study are publicly available on Kaggle. The training data were obtained from https://www.kaggle.com/datasets/andrewmvd/pulmonary-embolism-in-ct-images on 6 March 2025. The CIOF implementation and example scripts are available upon reasonable request from the corresponding authors via email.
